# Dr. Colin R. Tribe

**Published:** 1984-07

**Authors:** 


### Obituary

### C.R.T.

Most medical lives are spent being busy. When we are honest with ourselves, usually in the early hours of the morning, we know that we haven't given enough time to colleagues, wives, children and other members of our families.

Outside medicine we all meet friends who say as much, usually with exceptional politeness. A successful academic colleague, the son of a retired general medical practitioner, once said that he felt that medics were selected for their willingness to sacrifice almost everything to a demanding mistress—Medicine—who most times won over any wife or family.

Colin Tribe broke these rules. He was capable of overcoming this problem. Being busy, and taking time for informal chat were both possible for him. This is an exceptional gift.

His colleagues, by which one means his medical friends, were all charmed by this ability. We were deeply moved by Norma Brown's address[1] at Colin Tribe's Memorial Service. It must be no small tribute to Colin that so much was said by a close colleague on that occasion.

Few histopathological meetings in Bristol nowadays take place without the wish to consult Colin's opinion. We, all of us, live with a ghost. We remain greatly influenced by the views of a wise and gentle mentor. In so many ways, his pathological colleagues deeply mourn the passing of a personal friend.

### REFERENCE

1. N.J.B. (1984) Obituary: Dr. Colin Tribe. *Bristol Med.-Chi. J.* **99**, 61–63.

